# Pleomorphic spermatic cord liposarcoma: A case report and review of management

**DOI:** 10.1016/j.ijscr.2021.105725

**Published:** 2021-03-06

**Authors:** Babe Nejib Ebey, Sahbi Naouar, Bilel Faidi, Rayen Lahouar, Badreddine Ben Khalifa, Rafik El Kamel

**Affiliations:** a Urology Department, Les Aghlabides Surgical Division, Ibn Jazzar Teaching Hospital, Rue Hassan Ibn Nouaman, 3100, Kairouan, Tunisia; b Surgery Department, Les Aghlabides Surgical Division, Ibn Jazzar Teaching Hospital, Rue Hassan Ibn Nouaman, 3100, Kairouan, Tunisia

**Keywords:** Liposarcoma, Spermatic cord, Para testicular tumor, Orchiectomy

## Abstract

Liposarcoma of the spermatic cord is very rare, representing about 7% of para testicular sarcomas. It is considered to be one of the highest malignancy grades.

We present a case of a liposarcoma of the spermatic cord in a 45-year-old male complaining of a progressive painless swelling in the right inguinoscrotal region. Ultrasonography and computed tomography findings were compatible with liposarcoma of the spermatic cord. We performed a right radical orchiectomy with a wide resection of the mass. Histological examination confirmed the diagnosis and showed a pleomorphic subtype.

The mainstay of management of spermatic cord liposarcoma is wide excision with radical orchiectomy. The most important factors for prognosis are the histologic subtype and surgical margin status. Adjuvant radiotherapy should be considered in cases at high risk for local recurrence. Long-term surveillance is mandatory.

Liposarcoma of the spermatic cord is an uncommon para testicular tumor which should be part of the differential diagnosis of inguinoscrotal mass. A radical inguinal orchiectomy with wide resection of the soft tissue mass and the spermatic cord are the key to longest local and systemic disease-free survival.

## Highlights

• Pleomorphic liposarcoma of the spermatic cord is a very rare subtype of liposarcoma.
• It is considered to be one of the highest malignancy grades with high invasion, metastasis and recurrence.
• The diagnosis is a mixture of clinical, radiological and histological arguments.
• The mainstay of management is wide excision of the soft tissue mass with radical orchiectomy.
• The role of adjuvant treatments remains controversial.

## 1 Introduction

Liposarcomas are the most common of the soft tissue sarcomas, accounting for approximately 20% of all mesenchymal malignancies [1]. Liposarcoma of the spermatic cord (LSC) is very rare, representing about 7% of para testicular sarcomas [2]. They are frequently misdiagnosed after surgery for inguinal hernia, inguinal lymphadenectomy or testicular malignancy. Until now, the published literature on LSC has been limited to case reports with limited clinical information. To our knowledge only about 350 cases have been described in the literature. Pleomorphic liposarcoma is a rare subtype of liposarcoma and accounts for less than 5% of all Liposarcomas; it is considered to be one of the highest malignancy grades with high invasion, metastasis and recurrence [3]. We share our experience on the presentation of pleomorphic LSC in a 45 years-old male, and we review the management of this rare entity.

This procedure was performed by a junior resident with 5 years of specialized training and it has been reported in line with the SCARE 2020 criteria [4].

## 2 Presentation of case

A 45-year-old male with a medical history of right inguinal hernia surgery, presented to our department with a progressive painless swelling in the right inguinoscrotal region that was present for over 6 months, with rapid progression in last 2 months. There was no recent history of trauma, lower urinary tract symptoms, drug intake nor family history of cancer. On clinical examination, there was a firm and nodular scrotal mass. The right testis was not identified during palpation; the trans-illumination test was negative and there was no associated inguinal lymphadenopathy.

**Fig. 1:**
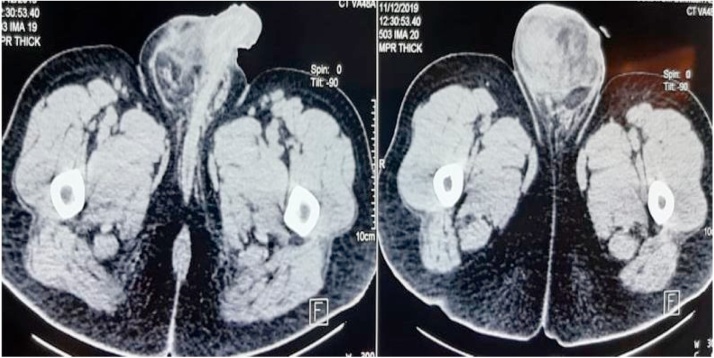
Axial CT Image showing a heterogeneously mass in the right hemi-scrotum, containing fat and soft tissue.

Ultrasonography (US) revealed a heterogeneous hyperechogenic mass in the right hemi-scrotum with mixed echogenicity. Computed tomography (CT) scan showed a 14 × 8 cm mass lesion in the right hemi-scrotum which contained fat and soft tissue (Fig. 1). The US and CT patterns were compatible with the fat-containing tumor, especially liposarcoma. Pre-operative imaging didn’t reveal any lymph node or distant metastasis. Testicular tumor markers, such as Alpha-fetoprotein, ß-human chorionic gonadotropin (ß-hCG), and lactate dehydrogenase (LDH) isoenzymes, were all normal.

Surgery was performed using a right inguinal approach. A yellowish soft tissue mass with a "bunch of grapes" appearance was closely attached to the spermatic cord; the testis was atrophic. Radical orchiectomy with wide resection of the mass was performed.

**Fig. 2:**
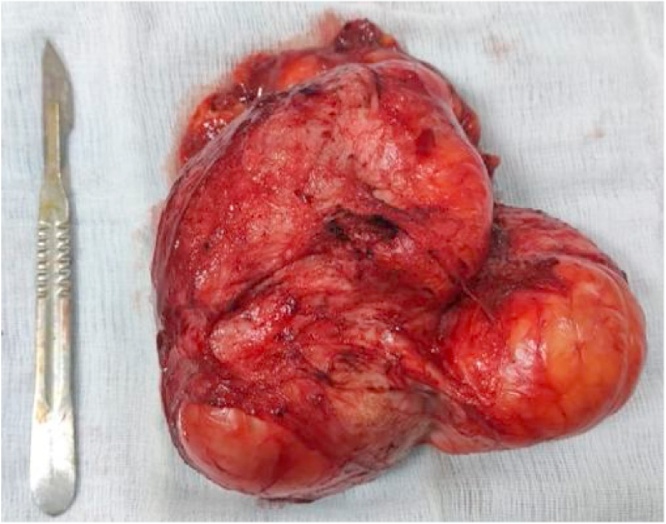
Macroscopic appearance of the surgical specimen showing a solid and nodular mass of the adipose tissue, encapsulated and attached to the spermatic cord.

**Fig. 3:**
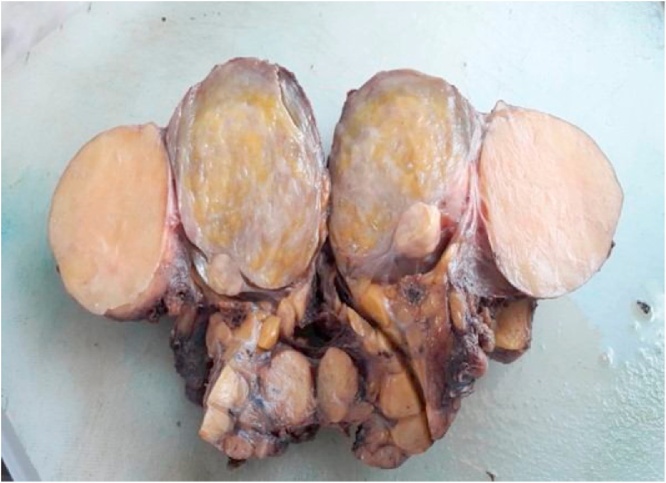
Gross examination of the mass revealed a large, encapsulated, yellow-brown colored fatty tissue. The mass weighed 736 gm. Atrophic testis was adherent to the mass.

Gross examination showed a 14 × 12 × 8 cm well-circumscribed, encapsulated and nodular mass (Fig. 2, Fig. 3). Microscopic examination revealed multinuclear giant lipoblasts with hyperchromatic nuclei and multivacuolated cytoplasm consistent with a pleomorphic subtype of LSC. The testis was atrophic, and it showed no pathological changes. The resected margins were negative and free of neoplastic cells.

The patient had a good postoperative clinical course with no complications and was discharged on the third postoperative day. Adjuvant radiotherapy was proposed; however, the patient refused any adjuvant treatment. Follow up with CT scans after 6 months were recommended. There have been no signs of any local recurrence during 1-year follow-up period.

## 3 Discussion

Liposarcomas of the spermatic cord are rare. They occur mostly in adults between 50 and 60 years [5]. They generally remain asymptomatic for years. The duration of symptoms, reported in the literature, ranged from 1 week to 5 years [6].

The typical clinical characteristics of LSC are painless, non-tender, nodular scrotal mass which progresses slowly and then increases rapidly in size [7]. In our case, total duration of symptoms was 6 months with rapid progression in last 2 months. Pain and tenderness have been reported in 10–15% of cases [8]. Rapid growth, large size, and symptomatic presentation are features suggestive of malignancy [8].

Sometimes the tumors are challenging to diagnose and are often mistaken as inguinal hernia, lipoma, hydrocele, epididymal cyst, or testicular tumors. Initially, inguinal and scrotal masses are evaluated by ultrasound in order to distinguish between intratesticular and extra testicular, solid and cystic masses [9]. Once the lesion is defined as solid, it is considered malignant until proven otherwise. CT scan is more useful for the diagnosis of liposarcoma, distinguishing tissue characteristics, morphologic features and tumor location and it determines the extent of the mass into the neighboring tissue [10]. However, there are no pathognomonic features for differentiating between benign and malignant masses. Magnetic resonance imaging (MRI) provides good information for the precise localization of the tumor and delineates its extension [11].

Tumor staging is based on histological examination and grading, and the presence of metastases. According to the World Health Organization’s International Classification of Disease, there are seven recognized classifications of liposarcoma: well, differentiated, myxoid, round cell, pleomorphic, mixed, dedifferentiated, and not otherwise specified [8]. Low-grade subtypes are histologically well-differentiated and have no metastatic potential, but lesions may recur locally. Local recurrence rate after conservative surgery alone reported in literature is up to 30%. High-grade subtypes (round-cell and pleomorphic) are rarer, but they are associated with a higher rate of recurrence and hematogenous metastasis to the lungs and bone [12]. The majority of cases of LSC reported in the literature were well-differentiated. In the present case, pathological examination of the specimen revealed pleomorphic subtype.

Due to its rarity, there is no gold standard treatment for LSC, including pleomorphic subtype. Radical orchiectomy, with wide excision of the soft tissue mass and excision of all potentially contaminated tissues from as close to the deep ring as possible, is the cornerstone of treatment of this neoplasm [7,12,13]. A positive surgical margin seems to be the main risk factor for early local recurrence and distant metastasis. Wide excision has demonstrated microscopic residual disease in 27% of apparently complete excision [14]. For this reason, the majority of reports acknowledge the importance of complete resection with clear microscopic margins and re-resection with wide resection in cases with positive margins in order to achieve a negative margin [12,13]. Routine lymph node dissection is currently not justified as the locoregional lymph nodes are rarely involved.

The use of adjuvant treatment (chemotherapy/radiotherapy) remains controversial, due to the paucity of data in the literature. The role of adjuvant radiotherapy is not clear. Two prospective randomized trials about the role of the addition of radiation to surgery in soft tissue sarcoma, showed that adjuvant radiotherapy can improve locoregional control and disease-free survival; however, this advantage did not translate into an overall survival benefit [2,12]. In another study, it has been demonstrated that postoperative radiotherapy can significantly decreased the 10-year local recurrence rate in patients with high-grade lesions (p = 0.0028) [15]. Therefore, adjuvant radiotherapy is recommended for high-grade tumors, lymphatic invasion, inadequate margins, or relapses [2,12,15]. Based on this limited literature, there is no definitive role for chemotherapy in the management of localized liposarcoma of the spermatic cord.

For patient follow-up, the consensus is to perform close and regular follow-up with imaging at 3, 6, 12 and 24 months [12]. These cancers have a known risk of local recurrence, hence a long-term follow-up up to 10 years is mandatory.

## 4 Conclusion

SCL is an uncommon paratesticular tumor which should be part of the differential diagnosis of inguino-scrotal mass. Up to now radical inguinal orchiectomy with wide resection of the soft tissue mass and the spermatic cord are the key to longest local and systemic disease-free survival. Lymph node dissection is not justified. The role of adjuvant radiotherapy remains controversial; however, it should be considered in cases at high risk for local recurrence, such as pleomorphic SCL. Chemotherapy has not proved its benefice until now.

## Declaration of Competing Interest

The authors report no declarations of interest.

## Funding

We have no sources of funding for our research.

## Ethical approval

This study is exempt from ethnical approval in our institution.

## Consent

Written informed consent was obtained from the patient for publication of this case report and accompanying images. A copy of the written consent is available for review by the Editor-in-Chief of this journal on request.

## Author contribution

Ebey Babe Nejib: writing the initial draft.

Sahbi Naouar: critical revision of the manuscript.

Bilel Faidi: critical revision of the manuscript.

Rayen Lahouar: writing of the final draft.

Badreddine Ben Khalifa: case report design.

Rafik El Kamel: final review.

## Registration of research studies

Not applicable.

## Guarantor

Professor El Kamel Rafik.

## Provenance and peer review

Not commissioned, externally peer-reviewed.
